# Pancreatic metastases from renal cell carcinoma: a case report and literature review of the clinical and radiological characteristics

**DOI:** 10.1186/1477-7819-11-289

**Published:** 2013-11-09

**Authors:** Yoshinori Hoshino, Hiroharu Shinozaki, Yuki Kimura, Yohei Masugi, Homare Ito, Toshiaki Terauchi, Masaru Kimata, Junji Furukawa, Kenji Kobayashi, Yoshiro Ogata

**Affiliations:** 1 Department of Surgery, Saiseikai Utsunomiya Hospital, 911-1 Takebayashi, Utsunomiya 321-0974, Japan; 2 Department of Pathology, Keio University, School of Medicine, 35 Shinanomachi, Tokyo 165-8582, Japan

**Keywords:** Pancreatic metastasis, Surgery, Renal cell carcinoma, Imaging, Radiological characteristics

## Abstract

Metastatic pancreatic cancer is rare, accounting for approximately 2% of all pancreatic malignancies, and most cases arise from renal cell carcinoma. We report the case of a 63-year-old woman, who presented with a pancreatic tumor detected during her annual health examination. She had undergone left nephrectomy 13 years previously for renal cell carcinoma. Computed tomography (CT) revealed two tumors in the head and body of the pancreas, a hypervascular tumor and a hypovascular tumor with an enhanced rim, respectively. She underwent pylorus-preserving pancreaticoduodenectomy, and metastatic pancreatic tumors arising from the kidney with clustered clear cell carcinoma immunohistochemically positive for CD10 were diagnosed. This report presents the different enhancement features of different lesions on CT scans. Because the enhancement features of lesions have been reported to vary according to the size of the metastatic tumor, a knowledge of the history of renal cell carcinoma is crucial for diagnosis.

## Background

Isolated metastasis to the pancreas is rare, ranging in incidence from 2% to 5% in clinical studies [1-6]. Renal cell carcinoma (RCC), melanoma, lung cancer, colorectal cancer and breast cancer are known to metastasize to the pancreas [7-11]. Most patients with pancreatic metastases are asymptomatic, whereas some exhibit jaundice or abdominal pain [12]. RCC has an annual incidence of more than 30,000 a year in the United States, and localized disease is treated via nephrectomy. Of patients with pancreatic metastases, 12% present with synchronous extrapancreatic metastasis, and they have a poor prognosis [13,14]. However, surgical treatment for isolated metachronous pancreatic metastases from RCC has been reported in recent years to improve prognosis [6,13-17]. In this study, we report a case of pancreatic metastases from RCC with different radiographic patterns for each lesion and review the radiographic patterns of pancreatic metastases using computed tomography (CT) and fluorodeoxyglucose (FDG)-positron emission tomography (PET).

## Case presentation

**Figure 1:**
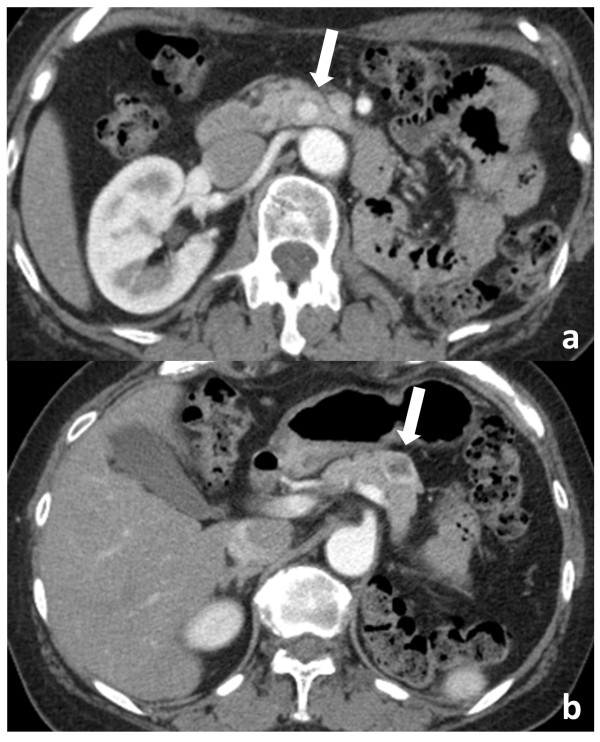
**CT findings of the pancreatic metastases (arrows). a**. Homogenous hypervascular head lesion. **b**. body lesion with central necrosis and enhanced rim.

**Figure 2:**
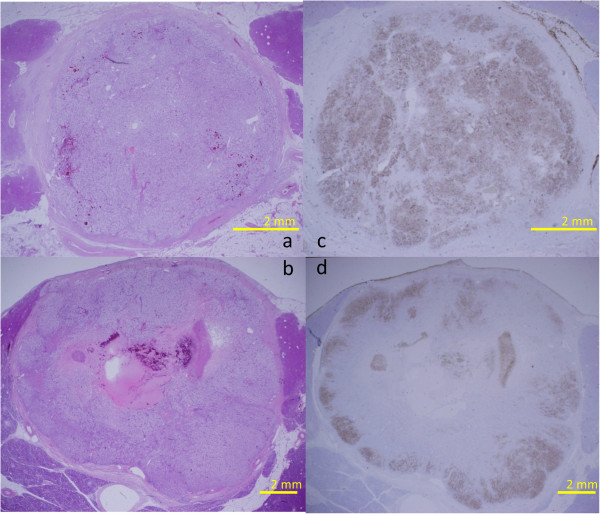
**Loupe images of resected specimen.** The head lesion **(a.c.)** consist of tumor cells, whereas the body with necreotic change in the center. **a**.**c**. Head lesion with hypervascular attenuation (**a**; H.E. stain, **c**; CD10). **b**.**d**. Body lesion with central hypodense (**b**; H.E. stain, **d**; CD10).

**Figure 3:**
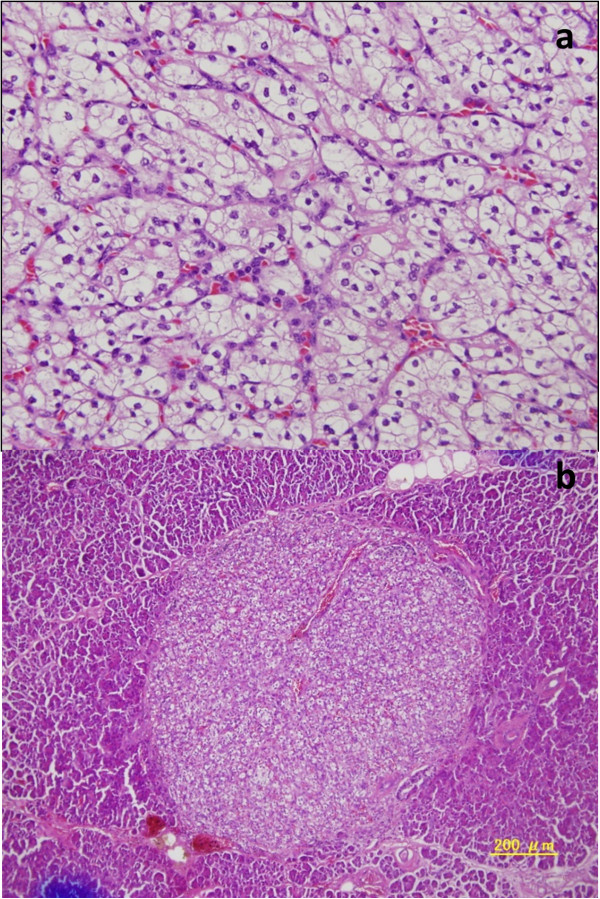
**Microscopic findings revealed clear cell carcinoma in all metastatic lesions including head, body and occult tumor. a**. Magnification of Head tumor with haematoxylin and eosin (H.E.) stain x100. **b**. metastatic tumor found occasionally in the head of the pancreas (1 mm) x40.

A 63-year-old woman had undergone left nephrectomy for RCC at our hospital 13 years previously. After 5 consecutive years of follow-up, she underwent an annual medical examination. Abdominal ultrasonography (US) revealed an abnormal mass in the body of the pancreas. CT revealed two lesions: a low-density mass (15 mm in diameter) in the pancreatic body that displayed rim enhancement and a homogeneously enhanced mass (8 mm in diameter) in the head (Figure 1). Magnetic resonance imaging (MRI) did not show enhancement in either lesion. FDG-PET did not show any abnormal metabolic activity in the pancreas. To allow a pathological diagnosis, endoscopic ultrasonography (EUS)-guided fine-needle aspiration biopsy was performed, but only necrotic tissue was obtained from the specimen. Although the radiographic features of the lesions were different, pancreatic metastases from RCC were strongly suspected because of the patient’s history of RCC. We noted the following from the laboratory findings: DUPAN-2, <25 U/ml (normal, <25 U/ml); Span-1, 2.3 U/ml (normal, <30 U/ml); carcinoembryonic antigen, 1.7 ng/ml (normal, <5.0 ng/ml); carbohydrate antigen 19-9, 2.6 U/ml (normal, <37 U/ml) and gastrin, 480 pg/ml (normal, <200 pg/ml). The patient underwent pylorus-preserving pancreaticoduodenectomy using the Imanaga method [18]. For the R0 resection, an extended pancreaticoduodenectomy was required rather than a classical resection. Intraoperative US revealed a low echoic mass with a bright halo and peripherally enriched blood flow in the body and a low echoic mass with homogeneously enriched blood flow in the head. Gross pathological examination revealed a 15 mm × 13 mm tumor occupying the body of the pancreas and another 8-mm tumor in the uncinate process of the pancreas. The head lesion was soft, whereas the body lesion was firm in consistency. The cut surface of the head lesion was yellow, whereas that of the lesion in the pancreatic body was grayish-white. Metastatic tumor cells homogeneously occupied the tumor in the head of the pancreas, and the firm lesion in the body of the pancreas showed a necrotic change in the center, which was surrounded by viable tumor cells and a fibrous capsule, identified as a low-density area on a CT scan (Figure 2). Immunohistochemically, the tumors were positive for CD10 and negative for chromogranin A and synaptophysin (Figure 2). Microscopic examination revealed large epithelial cells with clear cytoplasm and eosinophilic nuclei arranged in alveolar structures with abundant vascularity (Figure 3a). In addition, histological examination revealed another 1-mm occult micrometastatic lesion in the head of the pancreas (Figure 3b). The harvested lymph nodes and surgical margins were free of malignancy. Taken together, the pathological findings indicated that the lesions were metastases from RCC, and the thick enhanced rim of the body lesion was believed to be composed of viable RCC cells with high vascularity. The postoperative course was uneventful, and to date the patient has survived for 6 months without any evidence of recurrence or metastasis.

### Discussion

RCC accounts for approximately 2% of all adult malignances. Among kidney-limited diseases, RCC has a high overall survival rate (up to 95%) [19]. However, 20% to 30% of patients have metastases at presentation, and the 5-year survival rate is less than 10% once metastases spread [20]. In studies of resected specimens, RCC was the most common primary tumor leading to isolated pancreatic metastases [6,15]. RCC recurrence is classified as early or late recurrence. Late recurrence after nephrectomy is not common: recurrence is seen in 10% of patients after more than 10 years after surgery [21]. In most studies, the development of pancreatic metastasis was observed after a disease-free period after nephrectomy exceeding 10 years (maximum, 32.7 years) [22]. The relation between the metastatic location of the pancreas and the primary RCC is controversial [23] and can be either hematogeneous or via lymphatics [24]. Hematogeneous spread may occur along the draining collateral vein of a hypervascular renal tumor, and the spread through lymphatics may occur by retrograde lymph flow secondary to tumor infiltration of the retroperitoneal lymph nodes [25].

The symptoms of pancreatic metastasis are often nonspecific. Sellner *et al*. [13] reported in a review of 236 cases that 35% of patients were asymptomatic, whereas other patients had abdominal pain (20%), gastrointestinal tract bleeding (20%), obstructive jaundice (9%), weight loss (9%), pancreatitis (3%) or diabetes mellitus (3%). The diameter of the metastatic lesion was reported to have some association with patient symptoms. Bassi *et al*. [26] reported that the median tumor diameter in asymptomatic patients was 25 mm, compared to 45 mm in symptomatic patients. Conversely, Reddy *et al*. [27] reported that 93% (42/45) of patients had symptoms such as abdominal pain, jaundice or weight loss.

In general, the preoperative diagnosis of pancreatic metastases begins with a suspicion based on the patient’s history [28]. Imaging modalities such as CT, MRI, FDG-PET and EUS support the diagnosis. Muranaka *et al*. [29] reviewed the CT findings of pancreatic metastases from 28 metastatic carcinomas and classified these into 3 types according to their configuration: (1) a single localized metastasis (50% to 73%) [29-31]; (2) a diffuse enlargement with homogeneous attenuation of the pancreas (15% to 44%) [29-31] and (3) multifocal metastases (5% to 10%) [29-31]. Metastases from RCC are usually hypervascular and consequently display homogeneous contrast medium enhancement in the arterial phase of CT. Hyperenhancement of pancreatic metastases from RCC plays an important role in both the detection of tumor locations and the distinction of metastases from primary adenocarcinoma of the pancreas [32]. When hypervascular pancreatic tumors are identified on enhanced CT scans, differentiation from primary pancreatic endocrine tumors, intrapancreatic accessory spleens and vascular lesions is difficult. Palmowski *et al*. [4] observed two types of enhancement on CT scans of pancreatic metastases, namely lesions with either a homogeneous enhancement or a highlighted rim and nonenhancing internal components, depending on the size. In lesions greater than 15 mm in size, rim enhancement with hypodense central areas of necrosis has been observed on CT scans [32]. This hypodense aspect is associated with colonic metastases, hyperdense attenuation with RCC, or breast cancer [33,34]. We reviewed the radiological features of pancreatic metastases from RCC shown by dynamic CT scans (Table 1). Of 66 patients, 45 (68%) had homogeneous hypervascular enhancement features, whereas 21 (32%) had central hypovascularity with rim enhancement. Of these, 31 patients had a metastatic configuration, 5 patients had multifocal metastases to the pancreas and 26 patients had solitary tumors. No patients with pancreatic metastasis from RCC developed diffuse enlargement of the pancreas. In the present review, the size of pancreatic metastases from RCC has no particular relation with the presence of central hypodense areas because even tumors > 5 cm in size displayed hypervascular attenuation. The present case was classified as one with multifocal metastases in terms of the metastatic location. To our knowledge, this is the first case of concomitant multifocal metastases featuring two different enhancement characteristics, hypodense areas and homogenous hypervascularity.

**Table 1:** Radiologic features of pancreatic metastases from renal cell carcinoma shown by computed tomography

**Reference**	**Year**	**Number of patients**				
		**Enhancement features**			**Imaging configuration**	
		**Homogeneous hypervascularity**	**Central hypodense areas with rim enhancement**	**Total**	**Solitary**	**Multiple**
Marunaka *et al.*[29]	1989	1	0	1	1	0
Boudghene *et al.*[33]	1994	3	2	5	NA	NA
Scatarige *et al.*[35]	2001	1	1	2	2	0
Faure *et al.*[36]	2001	5	3	8	NA	NA
Yachida *et al.*[37]	2002	1	0	1	1	0
Zacharoulis *et al.*[38]	2003	3	0	3	2	1
David *et al.*[25]	2006	0	1	1	1	0
Palmowski *et al.*[4]	2008	12	10	22	NA	NA
Mecho *et al.*[39]	2009	4	2	6	6	0
You *et al.*[40]	2011	2	0	2	0	2
Katsourakis *et al.*[41]	2012	1	0	1	1	0
Atiq *et al.*[42]	2012	2	2	4	3	1
Comunoglu *et al.*[43]	2012	1	0	1	0	1
Yazbek *et al.*[44]	2012	9	0	9	9	0
**Totals**		**45**	**21**	**66**	**26**	**5**

NA, not available.

FDG-PET has not been established for the diagnosis of metastatic RCC. Ramdave *et al*. [45] reported that FDG-PET was useful for identifying distant metastases from RCC in all six of the patients in the study. Majhail *et al*. [46] calculated the sensitivity and specificity of FDG-PET for identifying distant metastases from RCC. They revealed that the sensitivity of the procedure was linked to the size of the metastases (83% for lesions larger than 15 mm; 93% for those larger than 20 mm). In the present patient with 15- and 8-mm lesions, FDG did not indicate any abnormal metabolic activity in either lesion.

Surgical resection of the pancreas is associated with substantial morbidity after surgery, and the survival benefit of surgery for metastatic lesions of the pancreas remains questionable since randomized control trials have not been conducted. However, surgical resection of metastatic deposits of RCC remains the most effective treatment because chemotherapy, immunotherapy and radiotherapy have generally proved to be ineffective for metastatic RCC [14,22,25,47]. We reviewed studies published from 1998 to 2013 that focused on surgical resection of the pancreas for metastases from RCC. The survival rates and features are listed in Table 2. The median 5-year overall survival rate after metastasectomy was reported to be 75% to 88%, compared to 0% to 50% without metastasectomy [13,14,17]. In a review, Masetti *et al*. [48] analyzed data for 159 patients who underwent metastasectomy of the pancreas for RCC: the median survival and 5-year survival rate were 5.8 years and 63.5%, respectively. Tanis *et al*. [49] reviewed 170 articles, and data for a total of 411 patients who underwent resection of pancreatic metastases were analyzed. They reported a pancreatic recurrence rate of 4.0% after a median of 42 months and an extrapancreatic recurrence rate of 17.1%. The 5-year survival rate was 72.6%. The surgical mortality rate after pancreaticoduodenectomy for pancreatic metastases was reported to be 2.6% [23]. Sellner *et al*. [13] compared the 5-year overall survival rate of patients with a solitary metastasis with those with multiple metastases (solitary metastasis, 64%; multiple metastases, 78%). Combining the findings outlined in several reports [50-52], radical surgery for multifocal metastases from RCC in the pancreas appears to be as justified as that for a solitary metastasis. Radical surgery with the R0 resection is the only curative option for patients with pancreatic metastases. Therefore, surgical resection for pancreatic metastases should be considered under individualized conditions including the management of comorbidities.

**Table 2:** Literature review of surgical treatment for pancreatic metastases from RCC

**Reference**	**Year**	**Number of patients**^**a**^	**Median age**	**Sex (F:M)**	**5-year survival**	**Median survival (months)**	**Time from primary treatment (months)**	**Median follow-up period (months)**
Butturini *et al.*[53]	1998	5	63	2:3	NA	24.5	120 (24–276)	19
Kassabian *et al.*[54]	2000	5	56	1:4	67%	NA	144 (48–180)	48
Ghavamian *et al.*[55]	2000	11	68	3:8	81%	120	108 (18–295)	50
Sohn *et al.*[17]	2001	10	63	4:6	80%	7	102 (0–336)	8
Faure *et al.*[36]	2001	8	57	2:6	88%	NA	83 (12–120)	38
Yachida *et al.*[37]	2002	5	60	2:3	100%	12	144 (36–288)	18
Law *et al.*[56]	2003	14	64	9:5	75%	NA	78 (0–300)	130
Wente *et al.*[47]	2005	15	63	10:5	NA	NA	85 (0–258)	10
Crippa *et al.*[6]	2006	5	65	3:2	80%	NA	36 (22–192)	41
Eidt *et al.*[15]	2007	7	64	2:5	88%	NA	160 (108–240)	36
Varker *et al.*[57]	2007	5	NA	NA	60%	NA	175	NA
Schauer *et al.*[58]	2008	10	62	5:5	60%	33	128 (5–277)	56
Zerbi *et al.*[14]	2008	23	64	15:8	88%	27	96 (12–276)	31
Reddy *et al.*[27]	2008	21	60	11:10	45%	58	110	over 120
Tanis *et al.*[49]	2009	10	63.5	2:8	NA	NA	107 (5–228)	NA
Masetti *et al.*[48]	2010	6	62	6:0	NA	NA	57.6 (20–288)	3
Konstantinidis *et al.*[59]	2010	20	68.5	7:13	61%	104	104	NA
Yazbek *et al.*[44]	2012	11	73	1:10	90%	84	136 (12–240)	NA
Gardini *et al.*[60]	2012	8	68	4:4	NA	NA	NA	38.6

NA: not available.

^a^Studies that included less than four patients were excluded.

## Conclusions

Multifocality of pancreatic metastasis has been reported to be in the range 20% to 45% [13,14]. In one report, a preoperative multifocality detection of 17.4% increased up to 34.8% on pathological examination of resected specimens [14]. Here, we report a rare case of pancreatic metastases with micrometastasis, which was not detected preoperatively, in the resected pancreas specimen on pathological examination. Moreover, pancreatic metastases from RCC can show both hypervascular attenuation and are central hypodense on CT scans although they are generally hypervascular tumors. Careful examination with multiple modalities for the diagnosis of the metastatic configuration and follow-up are recommended after surgery.

## Consent

Written informed consent was obtained from the patient for publication of this case report and any accompanying images. A copy of the written consent is available for review by the Editor-in-Chief of this journal.

## Abbreviations

CT: Computed tomography; EUS: Endoscopic ultrasonography; FDG: Fluorodeoxyglucose; HE: Hematoxylin and eosin; MRI: Magnetic resonance imaging; PET: Positron emission tomography; RCC: Renal cell carcinoma; US: Ultrasonography.

## Competing interests

The authors declare that they have no competing interests.

## Authors’ contributions

YH performed the majority of this study and drafted the manuscript. YK, HI, YM, and TT surveyed the literature. JF and MK critically revised the manuscript. HS, KK, and YO participated in the design and interpretation of this study under supervision. All authors read and approved the final manuscript.
